# Role of comorbidity on outcome of head and neck cancer: a population‐based study in Thuringia, Germany

**DOI:** 10.1002/cam4.882

**Published:** 2016-10-11

**Authors:** Irene Göllnitz, Johanna Inhestern, Thomas G. Wendt, Jens Buentzel, Dirk Esser, Daniel Böger, Andreas H. Mueller, Jörn‐Uwe Piesold, Stefan Schultze‐Mosgau, Ekkehard Eigendorff, Peter Schlattmann, Orlando Guntinas‐Lichius

**Affiliations:** ^1^ Department of OtorhinolaryngologyJena University HospitalJenaGermany; ^2^ Department of RadiooncologyJena University HospitalJenaGermany; ^3^ Department of OtorhinolaryngologySuedharzkrankenhaus NordhausenNordhausenGermany; ^4^ Department of OtorhinolaryngologyHelios‐Klinikum ErfurtErfurtGermany; ^5^ Department of OtorhinolaryngologySRH Zentralklinikum SuhlSuhlGermany; ^6^ Department of OtorhinolaryngologySRH Wald‐Klinikum GeraGeraGermany; ^7^ Department of Oromaxillofacial SurgeryHelios‐Klinikum ErfurtErfurtGermany; ^8^ Department of Oromaxillofacial Surgery and Plastic SurgeryJena University HospitalJenaGermany; ^9^ University Tumor CenterJena University HospitalJenaGermany; ^10^ Department of Medical StatisticsComputer Sciences and DocumentationJena University HospitalJenaGermany

**Keywords:** Cancer registry, Charlson comorbidity index, comorbidity, epidemiology, head and neck cancer, head and neck specific comorbidity index, risk, survival

## Abstract

To examine the impact of comorbidity on overall survival (OS) in a population‐based study of patients with head and neck cancer who were treated between 2009 and 2011. Data of 1094 patients with primary head and neck carcinomas without distant metastasis from the Thuringian cancer registries were evaluated concerning the influence of patient's characteristics and comorbidity on OS. Data on comorbidity prior to head and neck cancer diagnosis was adapted to the Charlson Comorbidity (CCI), age‐adjusted CCI (ACCI), head and neck CCI (HNCCI), simplified comorbidity score (SCS), and to the Adult Comorbidity Evaluation–27 (ACE‐27). Most patients were male (80%; median age: 60 years; 50% stage IV tumors). Smoking, alcohol abuse, and anemia were registered for 38%, 33%, and 23% of the patients, respectively. Predominant therapy was surgery + radiochemotherapy (30%), surgery (29%), and surgery + radiotherapy (21%). Mean CCI, ACCI, HNCCI, SCS and ACE‐27 were 1.0 ± 1.5, 2.6 ± 2.1, 0.6 ± 0.8, 4.4 ± 4.2, and 0.9 ± 0.9, respectively. Median follow‐up was 25.7 months. Multivariable analyses showed that higher age, higher UICC stage, no therapy, including surgery or radiotherapy, alcohol abuse, and anemia, higher comorbidity were independent risk factors for worse OS (all *P* < 0.05). According to the discriminatory power analysis none of the five comorbidity scores was superior to the other scores to prognosticate OS. This population‐based study showed that comorbidity is frequent in German patients with head and neck cancer and is an important risk factor for poor OS. Comorbidity should be routinely assessed and taken into account in prospective clinical trials.

## Introduction

The etiology of head and neck cancer still is primarily related to the classical risk factors smoking and alcohol abuse 1. Even many human papillomavirus (HPV)‐associated head cancer patients are active smokers at the time point of diagnosis with negative impact on survival 2. Smoking and alcohol consumption are also regarded as major causal factors for other chronic diseases like cardiovascular or pulmonary diseases and may therefore contribute to the comorbidity of patients with head and neck cancer 3. Furthermore, about half of the patients with newly diagnosed head and neck cancer are older than 60 years of age 4. Older age itself is correlated with higher comorbidity 5. Therefore, many patients with primary diagnosis of head and neck cancer have comorbidities that influence clinical decision making, treatment possibilities, and survival 6, 7, 8, 9, 10. Therefore, it is worthwhile to take comorbidity data into account when comparing patients' characteristics and treatment results between countries based on population‐based data 3. Interestingly, the impact of comorbidities on treatment decisions and survival in large and actual population‐based cohorts of head and neck cancer patients have so far been studied only in a few reports, for instance from Denmark 10, Netherlands 9, Taiwan 11, or the United States 5, 8, 12.

Therefore, we analyzed head and neck cancer patients diagnosed from 2009 to 2011 from the Cancer Registries in Thuringia to give representative epidemiological data on the impact of comorbidities on survival in Germany.

## Material and Methods

### Patients

The study was based on data of the Thuringian cancer registry database from 2009 to 2011. This is a population‐based registry collecting data from the five Thuringian cancer centers. These five databases (in the Thuringian towns: Nordhausen, Gera, Suhl, Jena, and Erfurt) register all cancer cases of the federal state Thuringia in the eastern part of Germany and cover about 98% of all patients with head and neck cancer in Thuringia 13.

**Figure 1:**
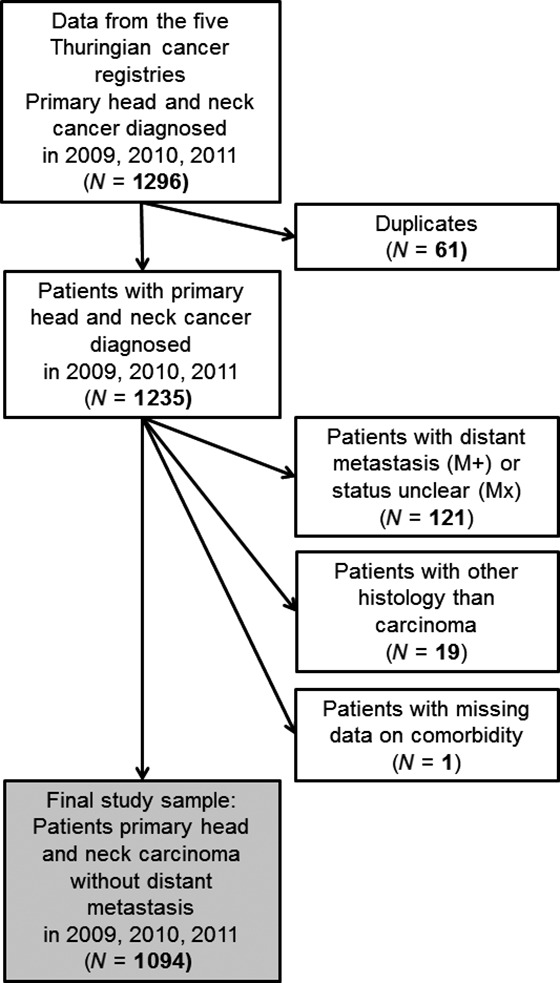
Flowchart of patient inclusion and exclusion.

New cases of head and neck cancer were classified according to the International Classification of Disease for Oncology (ICD‐O 14) and selected according to the following inclusion criteria: primary carcinoma of the head and neck region. Patients who were treated for recurrent disease only, skin cancer, other histologies (like lymphoma, sarcoma), metastasis in the head and neck region from other tumors sites were excluded. Duplicate records of patients have been removed. All cases with distant metastasis (M1) at primary diagnosis were excluded, too. The patient selection is summarized in Figure 1.

Extent of the disease was classified by pathological stages (pTNM) when appropriate, or clinical stages (cTNM) when a surgical resection was not performed, both according to the AJCC Cancer Staging Classification (2010). Because T or N classification were not clearly specified in all cases, stage grouping was not possible for 54 cases. Treatment presented in this presentation was defined as the first course of cancer‐specific treatment performed to treat the primary tumor and neck metastasis. Subsequent therapy to treat recurrences was not included in this definition of treatment.

### Comorbidity assessment

Information on preexisting comorbidity was derived from the secondary diagnoses coded according to International Classification of Diseases, 10th revision, German modification (ICD‐10‐GM), the patients' charts and drug plans. The index head and neck cancer was not coded as comorbidity. Four different comorbidity calculations were used. First, the Charlson comorbidity index (CCI) was utilized 15. The modification of the CCI for use with ICD‐10 codes as suggested by Sundararajan et al. was applied 16. The CCI is a weighted measure that incorporates 19 different medical categories and each weighted according to its potential to impact on mortality. Second, the age‐adjusted CCI (ACCI) was calculated 17. The ACCI scores were calculated with additional points added for age. The ACCI adds to the CCI score one point for each decade over the age of 40 of the patient. Third, the head and neck CCI (HNCCI) has been developed, especially for use in head and neck cancer patients 18. The HNCCI only used six out of the original 19 medical categories of the CCI and each identified conditions is assigned unweighted with one point each. Finally, the simplified comorbidity score (SCS) was calculated 19. The SCS was originally developed for patients with lung cancer. It is a weighted measure taking into account seven comorbid conditions including smoking. Patients were classified as smokers if they smoked cigarettes or quit smoking ≤3 months ago. All other patients were classified as nonsmokers. The ACE‐27 is a 27‐item comorbidity index for use with cancer patients 20. It grades specific diseases into three groups (grade 1 = mild, grade = moderate, grade 3 = severe) according to the severity of organ decompensation. Overall comorbidity score is defined according to the highest ranked single ailment, except in the case where two or more grade 2 ailments occur in different organ systems. In this situation, the overall comorbidity score is designated grade 3. Alcohol abuse was defined as more than one drink (14 g alcohol) per day, 7 per week, or binge drinking at diagnosis.

### Statistical analysis

To compare patients' characteristics as well as patients' therapy with their comorbidity, Pearson's chi‐square test for categorical data was used. If necessary scaled data were dichotomized to categorical data. The correlation with the different comorbidity scores was tested, using Spearman's correlation. Overall survival (OS) rates were calculated by the Kaplan–Meier method and differences of survival were compared by the log‐rank test. OS time was defined as the time of histological diagnosis to time of last follow‐up or death. Multivariable analyses were performed using the Cox proportional hazards model to estimate the hazard ratio (HR) with a confidence interval (CI) of 95% for overall survival including each comorbidity score separately, that is, five Cox models were calculated. Only significant parameters (*P* < 0.05) from the log‐rank tests were included into the multivariable analysis. Parameters included already into the calculation of comorbidity scores were not included once more into the multivariable analysis (i.e., age was not included in the multivariable analysis with the ACCI, the smoking parameter not in the analysis with the SCS, and the alcohol parameter was not included into the model for the ACI‐27 calculation). The performance of the models was assessed by evaluating calibration and discrimination. Discrimination denotes a model's ability to separate patients with events from those without events and was quantified, using Somer's D for censored data 21. Somer's D takes values between −1 and 1 and can be interpreted like the usual correlation coefficient. These values were internally validated using B = 150 bootstrap replications in order to avoid over optimistic results. Calibration referred to the extent of bias. Ideally, observed and predicted survival at 24 months after diagnosis would be identical. Here we used the difference between the observed and the predicted survival probability. Internal model validation was again performed using bootstrapping. This allowed reporting the mean calibration error and the corresponding 0.9 quantile. Model evaluation was performed with R and the package rms 22, 23. All other statistical analyses were with IBM SPSS version 23.0.0.0 statistical software for Windows (Chicago, IL). For all statistical tests, significance was two‐sided and set to *P* < 0.05.

## Results

### Patient's and tumor characteristics

1094 patients with primary head and neck cancer treated between 2009 and 2011 in Thuringia were included. 79.7% were male patients. Median age at diagnosis was 60.3 years. The distribution of the cases according to year of diagnosis, registry region, tumor site, staging, histology, grading, and therapy is shown in Table S1. Oropharynx (29.4%), oral cavity (27, 1%), and larynx (18.8%) were the predominant tumor sites. About half of the cases (49.7%) were diagnosed in tumor stage IV. Most carcinomas (88.0%) were squamous cell carcinomas. Major therapy strategies were surgery + radiochemotherapy (29.5%), surgery as monotherapy (29.1%), and surgery + radiotherapy (21.1%).

### Comorbidity

**Figure 2:**
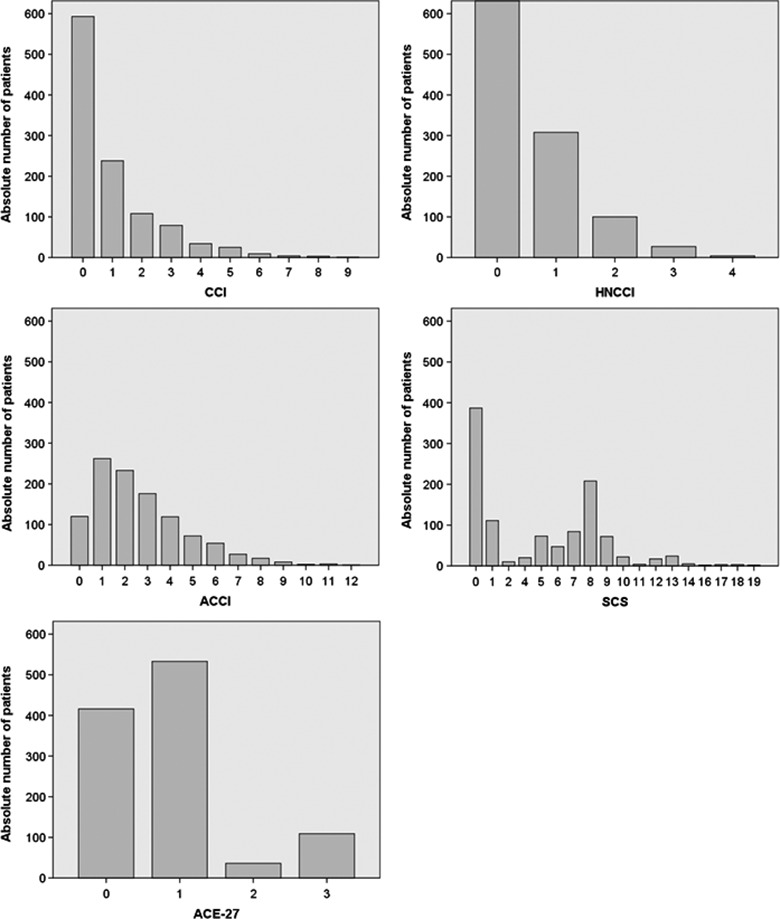
Distribution of comorbidity in the head and neck cancer population in Thuringia in 2009–2011 for different comorbidity scores. CCI, Charlson comorbidity index; ACCI, age‐adjusted CCI; HNCCI, revised head neck CCI; SCS, simplified comorbidity score; ACE‐27, adult comorbidity evaluation–27.

All comorbidity parameters are summarized in Table S2. Here 38% of the patients were smokers at the time point of diagnosis. Alcohol abuse was registered in 32.5%, 22.8% had an anemia according to the hemoglobin level at diagnosis. Median CCI was 0 (range: 0–9), median ACCI 2 (range: 0–12), median HNCCI 0 (0–4), median SCS was 5 (0–19), and median ACE‐27 was 1 (range: 0–3). The distribution of the scores in the entire patient collective is shown in Figure 2. The CCI and the derived indices ACCI and HNCCI showed a very high correlation (*r* = 0.860 and *r* = 0.813, respectively), The ACE‐27 correlated better with the SCS than with the CCI, ACCI, and HNCCI (*r* = 0.722, *r* = 0.509, *r* = 0.610), but also the SCS showed a high correlation with CCI, ACCI, and HNCCI (*r* = 301, *r* = 0.301, *r* = 0.455, respectively; Table S3). The SCS correlated with the ACE‐27, too (*r* = 0.665). The univariate analyses for associations between patients' characteristics (Table S4) as well as treatment characteristics (Table S5) to the comorbidity scores showed somewhat different results depending on which comorbidity calculation was considered. The CCI, HNCCI, ACE‐27 and SCS but not the ACCI showed significant gender differences. The CCI, HNCCI ad ACE‐27 but not the SCS showed differences between younger (<median of 60.3 years) and older patients (>median). Comorbidity was not different between early (stage I/II) and advanced disease (stage III/IV). Comorbidity (independent of the score used) was higher for alcohol drinkers and patients with anemia. Higher comorbidity was associated to higher probability to receive single modality treatment, less probability of receiving surgery or radiotherapy for the CCI, ACCI, and HNCCI but not for the SCS and the ACE‐27.

### Influence of comorbidity on overall survival

**Figure 3:**
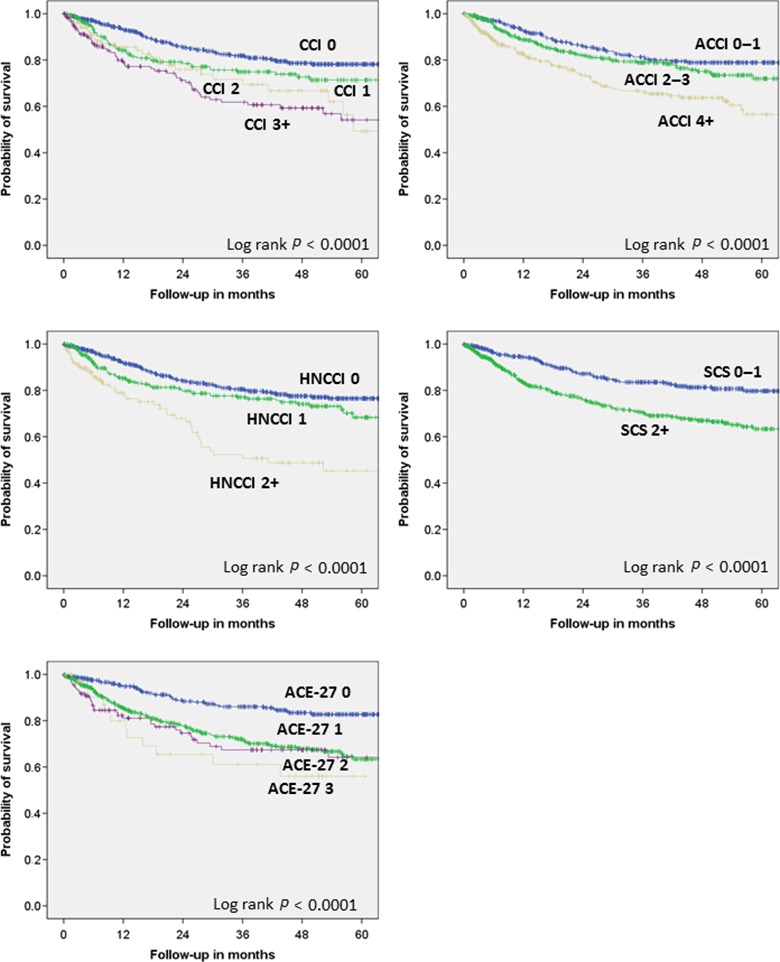
Kaplan–Meier curves on overall survival according to the five different comorbidity scores. CCI, Charlson comorbidity index; ACCI, age‐adjusted CCI; HNCCI, revised head neck CCI; SCS, simplified comorbidity score; ACE‐27, adult comorbidity evaluation–27.

Eight hundred‐seventy five (80%) patients were alive and 219 (20%) patients were dead. Median follow‐up was 25.7 months. Median follow‐up of patients alive was 34.4 months. Of all patients, 656 (60.0%) were recurrence free, and 428 (40.8%) had developed a tumor recurrence. From the patients alive, 604 (69.0%) were recurrence free, and 271 (31.0%) had developed a tumor recurrence during follow‐up. The univariate analyses showed that several baseline and treatments characteristics and as well as the comorbidity were significantly associated to lower OS (Table S6): Higher age, other site than larynx, advanced stage (III/IV), squamous cell carcinoma, no surgery as part of the treatment concept, no radiotherapy, chemotherapy, only best supportive care, smoking, drinking anemia, CCI 2 + , ACCI 4 + , HNCCI 2 + , SCS 2 + , and ACE‐27 2–3 were all related to lower overall survival (all *P* < 0.05). A closer examination of different classification subgroups of the four comorbidity indices (Fig. 3) showed a good separation of the survival curves between CCI 0, CCI 1/2 and CCI 3 + . ACCI 0–1 was clearly separated from ACCI 4 +  but not from ACCI 2–3. Patients with HNCCI 0 showed a distinctly separated OS curve from patients with HNCCI 2 +  but the difference to HNCCI 1 was low. SCS 0–1 patients showed a much better OS than SCS 2 + . This separation (SCS 1 versus SCS 3 + ) was much more distinct than the often applied separation between SCS 0–9 to SCS 10 +  (*P* < 0.0001 versus *P* = 0.010, respectively). The Kaplan–Meier curve was at best separated between the group of patients with ACE‐27 0 and the other ACE‐27 curves (grade 1, 2, 3).

Five different multivariable analyses were performed, separately including one of the four comorbidity indices (Table 1, 2, 3). Higher age, higher stage, alcohol consumption at diagnosis, anemia at diagnosis, no surgery, no radiotherapy and all four comorbidity indices were independent predictors for worse OS. Increasing CCI score was independently associated with increased risk of death after 5 years with adjusted hazard ratio (HR) of HR = 1.23 (95% confidence interval [CI] = 0.86–1.75) for CCI 1, HR = 1.66 (CI = 1.02–2.71) for CCI 2 and HR = 1.87 (CI = 1.87–2.74) for CCI 3 + , respectively, compared to CCI 0. ACCI 2–3 had a HR = 1.32 (CI = 0.93–1.86), ACCI 4 +  a HR = 2.67 (CI = 1.65–3.39), respectively, compared to ACCI 0–1. HNCCI 1 showed a HR = 1.07 (CI = 0.77–1.48), and HNCCI 2 +  of HR = 2.08 (CI = 1.40–3.09), respectively, compared to HNCCI 0. Finally, a SCS 2 +  had a HR = 1.50 (CI = 1.08–2.07) compared to SCS 0–1. ACE‐27 grade 1 had a HR = 1.79 (CI = 1.25–2.56), ACE‐27 grade 2 a HR = 1.793 (CI = 1.25–2.56), and a ACE‐27 grade 3 a HR = 1.88 (CI = 1.15–3.06), compared to patients with ACE‐27 grade 0. The ACE‐27 was therefore the only comorbidity score where higher comorbidity did not show a continuous increase of the HR.

**Table 1:** Multivariable Cox regression models of risk factors for overall survival including the CCI or ACCI comorbidity score

Factor	HR	95% CI		*P*		HR	95% CI		*P*
		Lower	Upper				Lower	Upper	
Comorbidity									
CCI 0	1			**0.009**	ACCI 0‐1	1			**<0.0001**
CCI 1	1.227	0.860	1.750	0.260	ACCI 2‐3	1.320	0.929	1.875	0.121
CCI 2	1.666	1.023	2.711	**0.040**	ACCI 4+	2.367	1.654	3.388	**<0.0001**
CCI 3+	1.872	1.279	2.740	**0.001**					
Age									
<50 years	1			**0.013**		NA			
50–59 years	1.239	0.775	1.980	0.371					
60–69 years	1.621	0.996	2.638	0.052					
70–79 years	1.993	1.157	3.432	**0.013**					
80 + years	3.484	1.573	7.717	**0.002**					
Site									
Larynx	1					1			
Other	1.303	0.872	1.948	0.197		1.248	0.838	1.860	0.276
UICC stage									
I	1			**<0.0001**		1			**<0.0001**
II	3.563	1.856	6.837	**<0.0001**		3.422	1.780	6.579	**<0.0001**
III	4.651	2.370	9.127	**<0.0001**		4.643	2.365	9.115	**<0.0001**
IV	5.716	3.093	10.562	**<0.0001**		5.712	3.090	10.558	**<0.0001**
Histology									
Other	1					1			
SCC	1.497	0.843	2.661	0.169		1.476	0.834	2.613	0.182
Surgery									
Yes	1					1			
No	2.064	1.481	2.876	**<0.0001**		2.099	1.504	2.928	**<0.0001**
Radiotherapy									
Yes	1					1			
No	1.881	1.258	2.813	**0.002**		1.969	1.320	2.939	**0.001**
Chemotherapy									
Yes	1					1			
No	0.818	0.577	1.160	0.260		0.869	0.619	1.222	0.420
Smoker									
No	1					1			
Yes	1.215	0.857	1.724	0.274		1.146	0.813	1.616	0.436
Alcohol									
No	1					1			
Yes	1.689	1.183	2.413	**0.004**		1.620	1.144	2.295	**0.007**
Anemia									
No	1					1			
Yes	1.396	1.024	1.903	**0.035**		1.456	1.072	1.977	**0.016**

CCI, Charlson comorbidity index; ACCI, Age‐adjusted CCI; HR, Hazard ratio; CI, confidence interval; SCC, squamous cell carcinoma; NA, not applicable. Significant *p*‐values (*p*<0.05) in bold.

**Table 2:** Multivariable Cox regression models of risk factors for overall survival including the HNCCI or SCS comorbidity score

Factor	HR	95% CI		*P*		HR	95%CI		*P*
		Lower	Upper				Lower	Upper	
Comorbidity									
HNCCI 0	1			**0.001**	SCS 0‐1	1			
HNCCI 1	1.069	0.772	1.479	0.689	SCS 2+	1.496	1.080	2.073	**0.015**
HNCCI 2+	2.081	1.403	3.087	**<0.0001**					
Age									
<50 years	1			**0.005**		1			**0.001**
50–59 years	1.269	0.795	2.028	0.318		1.260	0.790	2.009	0.331
60–69 years	1.681	1.036	2.727	**0.036**		1.788	1.107	2.889	**0.018**
70–79 years	2.073	1.206	3.561	**0.008**		2.196	1.290	3.737	**0.004**
80 + years	3.892	1.765	8.583	**0.001**		4.094	1.886	8.886	**<0.0001**
Site									
Larynx	1					1			
Other	1.286	0.863	1.916	0.217		1.245	0.835	1.855	0.282
UICC stage									
I	1			**<0.0001**		1			**<0.0001**
II	3.351	1.741	6.448	**<0.0001**		3.677	1.918	7.050	**<0.0001**
III	4.513	2.298	8.864	**<0.0001**		4.812	2.460	9.413	**<0.0001**
IV	5.383	2.913	9.950	**<0.0001**		5.883	3.182	10.877	**<0.0001**
Histology									
Other	1					1			
SCC	1.536	0.864	2.730	0.144		1.547	0.870	2.750	0.137
Surgery									
Yes	1					1			
No	2.102	1.504	2.937	**<0.0001**		2.158	1.551	3.001	**<0.0001**
Radiotherapy									
Yes	1					1			
No	1.853	1.236	2.778	**0.003**		1.943	1.300	2.903	**0.001**
Chemotherapy									
Yes	1					1			
No	0.803	0.565	1.139	0.219		0.863	0.608	1.224	0.409
Smoker									
No	1					NA			
Yes	1.212	0.854	1.721	0.282					
Alcohol									
No	1					1			
Yes	1.675	1.173	2.392	**0.005**		1.667	1.205	2.306	**0.002**
Anemia									
No	1					1			
Yes	1.427	1.047	1.945	**0.024**		1.461	1.077	1.981	**0.015**

HNCCI, Head and neck CCI; SCS, Simplified Comorbidity Score; HR, Hazard ratio; CI, confidence interval; SCC, squamous cell carcinoma; NA, not applicable.

**Table 3:** Multivariable Cox regression models of risk factors for overall survival including the ACE‐27 comorbidity score

Factor	HR	95% CI		*P*
		Lower	Upper	
Comorbidity				
ACE‐27 grade 0	1			**0.004**
ACE‐27 grade 1	1.793	1.253	2.564	**0.001**
ACE‐27 grade 2	2.464	1.258	4.826	**0.009**
ACE‐27 grade 3	1.875	1.148	3.062	**0.012**
Age				
<50 years	1			**0.008**
50–59 years	1.234	.774	1.967	.377
60–69 years	1.673	1.039	2.694	**0.034**
70–79 years	1.987	1.173	3.367	**0.011**
80 + years	3.370	1.556	7.299	**0.002**
Site				
Larynx				
Other	1.286	.863	1.917	0.217
UICC stage				
I	1			**<0.0001**
II	3.568	1.857	6.858	**<0.0001**
III	4.662	2.373	9.159	**<0.0001**
IV	5.757	3.116	10.638	**<0.0001**
Histology				
Other	1			
SCC	1.578	0.887	2.806	0.121
Surgery				
Yes	1			
No	2.135	1.534	2.973	**<0.0001**
Radiotherapy				
Yes	1			
No	1.870	1.253	2.790	**0.002**
Chemotherapy				
Yes	1			
No	0.849	0.600	1.201	0.354
Smoker				
No	1			
Yes	1.407	1.034	1.913	**0.030**
Alcohol				
No	NA			
Yes				
Anemia				
No	1			
Yes	1.469	1.083	1.994	**0.014**

ACE‐27, adult comorbidity evaluation–27; HR, hazard ratio; CI, confidence interval; SCC, squamous cell carcinoma; NA, not applicable.

The results of the comparison of the discriminatory ability of the five comorbidity scores to differentiate between patients alive or dead 24 months after diagnosis is shown in Table 4. Somer's D and corrected Somer's D were not substantially different between the five comorbidity scores. The difference between the observed and the predicted survival probability was also low independently of the comorbidity score chosen for multivariable analysis. The likelihood ratio test showed that all five comorbidity tests were able to improve the fit of the multivariable regression models shown in Table 1, 2, 3.

**Table 4:** Comparison of the performance of the five multivariable models including the comorbidity indices in comparison to multivariable analysis without inclusion of a comorbidity index

Comorbidity index	Somer's D	Corrected Somer's D	Mean calibration error	0.9 percentile	Likelihood ratio test	*P*
None^a^	0.4634	0.4239	0.016	0.009		
CCI	0.4776	0.4323	0.017	0.009	14.09	0.0028
ACCI	0.4663	0.4337	0.014	0.007	23.37	0.0001
HNCCI	0.4741	0.4309	0.018	0.009	15.44	0.0004
SCS	0.4741	0.4382	0.018	0.011	5.49	0.0190
ACE‐27	0.4812	0.4405	0.018	0.010	8.76	0.0327

a Multivariable analysis, including the parameters age, site, UICC stage, histology, surgery, radiotherapy, chemotherapy, smoker, alcohol, and anemia.

CCI, Charlson comorbidity index; ACCI, Age‐adjusted CCI; HNCCI, head and neck CCI; SCS, Simplified comorbidity score; ACE‐27, adult comorbidity evaluation–27.

## Discussion

This population‐based study verified the importance of comorbidity in German patients with head and neck cancer. Depending on which of the four applied comorbidity indices was used, it could be shown that 40.1–65.1% of the patients had an important comorbidity. The degree of comorbidity was independent from other risk factors associated with worse overall survival.

This is the first large study in which the occurrence of comorbidities in head and neck cancer patients has been studied in a federal state of Germany. The Free State of Thuringia is a small territorial area with currently 2.2 million habitants. The number of hospitals treating head and neck cancer is transparent: Eight department of otorhinolaryngology and two departments of maxillofacial surgery are treating these patients and the data is delivered to five cancer registries 4. In contrast, most knowledge on comorbidity of head and neck cancer patients, using standardized and validated comorbidity scores is founded on hospital‐based studies, that is, data from large tertiary institutions typically collected over many years in small or undefined populations based on the allocation area of the hospital (actual overview on hospital‐based data in the review from Boje et al. 24 and in Table S7 and S8). Such a referral and/or selection bias might explain why the number of head and neck cancer patients with comorbidities varies in hospital‐based studies between 18% and 81%.

Only a few comparable and actual epidemiological population‐based studies on the role of comorbidity on OS in head and neck cancer patients from the United States, Taiwan, Denmark, and Netherlands have been published 5, 8, 9, 10, 11, 20, 25, 26. In these studies, the incidence of comorbidity varied between 12% and 65% when using the CCI, that is, some studies revealed higher and other lower comorbidity rates than in the present study with 46%. Only Piccirillo et al 20 used the ACE‐27 and revealed an incidence of 45%, that is, lower than in the present study with 62%. One might only conclude that comorbidity of head and neck cancer patients seems to be lower than in European cohorts. Using the HNCCI, the comorbidity incidence of the present study with 40% was higher than in the only other two studies, using the HNCCI with incidence of 27% in the Danish study and 25% in the study from Taiwan 11, 18. There was only one population‐based study using the ACCI and showed a lower comorbidity incidence in Taiwan with 60% than in the present study with 89% 11. This study was restricted to nasopharyngeal cancer and these patients were much younger and might directly explain the differences when calculating the comorbidity based on the ACCI. In general, although all mentioned studies were population‐based, a direct comparison was difficult, because analyzed subsites, tumor stages, and types of therapy varied. Hence, it cannot be answered why comorbidity is such different between the mentioned studies and compared to the present study. Two of these studies (like the present study) were not restricted to one subsite, included all age groups, and analyzed only patients without distant metastasis (M0) 8, 18. Also Rose et al. showed that comorbidity is an independent risk factor for survival 8, but the Danish study by Boje et al. also using comorbidity indices (CCI and HNCCI). The present German cohort had a higher comorbidity than the Danish cohort regardless of the index used. Both studies are comparable due to the median age and gender distribution of the cohorts. Unfortunately, no data on tobacco and alcohol consumption is given for the Danish cohort. It is generally accepted that tobacco and alcohol abuse are not only major risk factors for the development of head and neck cancer, but also are major risk factors for many comorbidities and explain in large part of the excess mortality due to causes other than head and neck cancer 27. Surprisingly, beyond the present study only one other population‐based study presented data on tobacco and alcohol abuse: In the study by Genther et al., restricting patients older than 65 years of age, 12% of the patients were current smokers and 5% had alcohol abuse as a comorbid illness 5. Also in other actual non‐population‐based studies from the Unites States including all age cohorts, the relative portions of current smokers comprised <15% 2, 28. In present study, the incidence of tobacco and alcohol abuse was 38 and 33%, respectively, and for patients older than 65 years of age 21% and 19%, respectively. This is reflected in the higher incidence of tobacco and alcohol abuse in the German population and in German patients with head and neck cancer compared to the United States 29. We assume that the relative high comorbidity in the present study compared to data from other countries is directly related to the high incidence of smokers and alcohol drinkers.

Which comorbidity index is now the best? All comorbidity scores revealed that comorbidity was an independent risk factor for worse overall survival, but a better discrimination of any of the five applied scores was not seen. We interpret this as a meaning to use routinely a validated comorbidity score, but which one is chosen does not matter. Higher comorbidity was significantly associated with higher risk of death for all scores but the ACE‐27. Patients with ACE‐27 grade 2 paradoxically had a higher risk (HR = 2.464) than patients with ACE‐27 grade 3 (HR = 1.875). We cannot give an explanation for this finding.

None of the applied comorbidity indices includes anemia or the hemoglobin level, although is well known and was confirmed by the present study that anemia is an important risk factor for worse overall survival 30, 31. It might be worthwhile to analyze if the additional implementation of anemia into a comorbidity index allows an improvement of the discriminatory power of different comorbidity grades.

Population‐based studies based on cancer registry data have several limitations. Many cancer registries like the German registries do not collect systematically data on comorbidity, smoking and drinking habits. To overcome this limitation, we analyzed the patients' charts. The accuracy of the comorbidity assessment depends on the quality of disease coding in the charts. It might be that the comorbidity was underestimated due to missing data. A patient‐reported comorbidity questionnaire filled out at diagnosis would perhaps be helpful to improve the comorbidity assessment 32. We could not evaluate one important risk factor: HPV is not regularly assessed in all new patients with head and neck cancer in Thuringia. Comorbidity seems to be an independent prognostic factor also in HPV‐positive tumors 33. Furthermore, the registries do not contain data on the process of treatment decision. Therefore, it was not possible to analyze the effect of comorbidity on treatment decision. It might be that comorbidity had an influence on individuals and may be nonstandard treatment decisions 34.

The National Comprehensive Cancer Network (NCCN) guidelines for head and neck cancer (Version I.2015; www.NCCN.org) state that documentation of comorbidity is important to facilitate optimal treatment selection. European guidelines (for instance:^35^) even do not mention comorbidity as an important factor. It would be desirable to develop more precise rules in the guidelines as to how comorbidity should influence treatment decisions. Furthermore, comorbidity assessment should finally become an integral part of risk stratification in clinical trials 3. Finally, to facilitate the implementation of comorbidity assessment in clinical routine of head and neck cancer treatment, modern electronic health care database systems should be used to make comorbidity assessment more effective 36, and modern tools like electronic applications for rapid calculation of comorbidity scores should be used 37.

In conclusion, the presented population‐based study showed that the incidence of comorbidity is high in German patients with head and neck cancer independent of the tumor stage. We recommend using routinely a validated comorbidity score when calculating the probability of survival of head and neck cancer patients. It would be desirable when comorbidity data would be recorded routinely in head and neck cancer registries.

## Conflict of Interest

None declared.

## Supporting information


**Table S1.** Distribution of Thuringian head and neck cancer patients diagnosed in 2009–2011 according to clinical and demographic parameters (*N* = 1094 patients).
**Table S2.** Comorbidity of Thuringian head and neck cancer patients diagnosed in 2009 to 2011 (*N* = 1094 patients).
**Table S3.** Correlation with the different comorbidity scores.
**Table S4.** Association between patients' characteristics and comorbidity.
**Table S5.** Association between patients' therapy and comorbidity.
**Table S6.** Association of baseline parameters and of comorbidity on overall survival (*N* = 1094 patients).
**Table S7.** Comparison of the present study to other population‐based studies on the impact of comorbidity on overall survival in head and neck cancer.
**Table S8.** Comparison of the present study to other larger hospital‐based studies (>100 patients) on the impact of comorbidity on overall survival in head and neck cancer.
