# Supplementary material for: Role of comorbidity on outcome of head and neck cancer: a population‐based study in Thuringia, Germany
Source: Cancer Med. 2016 Oct 11;5(11):3260–71. doi: 10.1002/cam4.882 (PMC5119982; doi:10.1002/cam4.882)
Supplement: Supplementary file 1 — Table S1. Distribution of Thuringian head and neck cancer patients diagnosed in 2009–2011 according to clinical and demographic parameters (N = 1094 patients). Table S2. Comorbidity of Thuringian head and neck cancer patients diagnosed in 2009 to 2011 (N = 1094 patients). Table S3. Correlation with the different comorbidity scores. Table S4. Association between patients' characteristics and comorbidity. Table S5. Association between patients' therapy and comorbidity. Table S6. Association of baseline parameters and of comorbidity on overall survival (N = 1094 patients). Table S7. Comparison of the present study to other population‐based studies on the impact of comorbidity on overall survival in head and neck cancer. Table S8. Comparison of the present study to other larger hospital‐based studies (>100 patients) on the impact of comorbidity on overall survival in head and neck cancer. [file CAM4-5-3260-s001.docx]

**Supplemental Tables**

**Supplemental Table 1**

| **Supplemental Table 1.** Distribution of Thuringian head and neck cancer patients diagnosed in 2009 to 2011 according to clinical and demographic parameters (N = 1,094 patients) | | |
| --- | --- | --- |
| Parameter | Frequency (N) | % |
| Year of diagnosis |  |  |
| 2009 | 352 | 32.2 |
| 2010 | 357 | 32.6 |
| 2011 | 385 | 35.2 |
| Tumor registry region |  |  |
| Erfurt | 391 | 35.7 |
| Jena | 323 | 29.5 |
| Gera | 195 | 17.8 |
| Nordhausen | 94 | 8.6 |
| Suhl | 91 | 8.3 |
| Gender |  |  |
| Male | 872 | 79.7 |
| Female | 222 | 20.3 |
| Age cohorts |  |  |
| <50 years | 160 | 14.6 |
| 50-59 years | 380 | 34.7 |
| 60-69 years | 281 | 25.7 |
| 70-79 years | 224 | 20.5 |
| 80+ years | 49 | 4.5 |
| Site |  |  |
| Lip | 23 | 2.1 |
| Oral cavity | 296 | 27.1 |
| Oropharynx | 322 | 29.4 |
| Nasopharynx | 22 | 2.0 |
| Hypopharynx | 113 | 10.3 |
| Larynx | 206 | 18.8 |
| Nose and paranasal sinus | 40 | 3.7 |
| Salivary glands | 70 | 6.4 |
| Middle ear | 2 | .2 |
| Stage |  |  |
| I | 221 | 20.2 |
| II | 130 | 11.9 |
| III | 145 | 13.3 |
| IV | 544 | 49.7 |
| unstaged (TX and/or NX) | 54 | 4.9 |
| Histology |  |  |
| Squamous cell carcinoma | 963 | 88.0 |
| Other carcinoma | 131 | 22.0 |
| Grading |  |  |
| G1/G2 | 692 | 63.3 |
| G3/G4 | 292 | 26.7 |
| GX | 110 | 10.1 |
| Therapy |  |  |
| Surgery | 318 | 29.1 |
| Surgery + radiochemotherapy | 323 | 29.5 |
| Surgery + radiotherapy | 231 | 21.1 |
| Surgery + radiotherapy + cetuximab | 30 | 2.7 |
| Radiotherapy | 41 | 3.7 |
| Radiochemotherapy +/- cetuximab | 108 | 9.9 |
| Radiotherapy + cetuximab | 12 | 1.1 |
| Chemotherapy and/or cetuximab | 6 | 0.5 |
| Best supportive care | 25 | 2.3 |

**Supplemental Table 2**

| **Supplemental Table 2.** Comorbidity of Thuringian head and neck cancer patients diagnosed in 2009 to 2011 (N = 1,094 patients) | | |
| --- | --- | --- |
| Parameter | Frequency (N) | % |
| Smoking |  |  |
| No | 678 | 62.0 |
| Yes | 416 | 38.0 |
| Alcohol |  |  |
| No | 738 | 67.5 |
| Yes | 356 | 32.5 |
| Anemia |  |  |
| No (Hemoglobin level normal) | 827 | 75.6 |
| Yes (Hemoglobin level below norm) | 249 | 22.8 |
| Unknown | 18 | 1.6 |
| Charlson comorbidity index (CCI) |  |  |
| CCI 0 | 593 | 54.2 |
| CCI 1 | 238 | 21.8 |
| CCI 2 | 108 | 9.9 |
| CCI 3+ | 155 | 14.2 |
| Age-adjusted CCI (ACCI) |  |  |
| ACCI 0-1 | 382 | 34.9 |
| ACCI 2-3 | 409 | 37.4 |
| ACCI 4+ | 303 | 27.7 |
| Head and neck CCI (HNCCI) |  |  |
| HNCCI 0 | 655 | 59.9 |
| HNCCI 1 | 308 | 28.2 |
| HNCCI 2+ | 131 | 12.0 |
| Simplified Comorbidity Score (SCS) |  |  |
| SCS 0-1 | 498 | 45.5 |
| SCS 2+ | 596 | 54.5 |
| Adult Comorbidity Evaluation–27 (ACE-27) |  |  |
| ACE-27 grade 0 | 416 | 38.0 |
| ACE-27 grade 1 | 533 | 48.7 |
| ACE-27 grade 2 | 36 | 3.3 |
| ACE-27 grade 3 | 109 | 10.0 |

**Supplemental Table 3**

| **Supplemental Table 3.** Correlation between the different comorbidity scores. | | | | | |
| --- | --- | --- | --- | --- | --- |
|  | **CCI** | **ACCI** | **HNCCI** | **SCS** | **ACE-27** |
| **CCI** | --- | r=0.860, p<0.001 | r=0.813; p<0.001 | r=0.453; p<0.001 | r=0.722; p<0.001 |
| **ACCI** | r=0.860, p<0.001 | --- | r=0.713; p<0.001 | r=0.301; p<0.001 | r=0.509; p<0.001 |
| **HNCCI** | r=0.813; p<0.001 | r=0.713; p<0.001 | --- | r=0.455; p<0.001 | r=0.610; p<0.001 |
| **SCS** | r=0.453; p<0.001 | r=0.301; p<0.001 | r=0.455; p<0.001 | --- | r=0.665; p<0.001 |
| **ACE-27** | r=0.722; p<0.001 | r=0.509; p<0.001 | r=0.610; p<0.001 | r=0.665; p<0.001 | --- |

CCI = Charlson Comorbidity Index; ACCI = age-adjusted CCI; HNCCI = Head neck CCI; SCS = simplified comorbidity scale; ACE-27 = Adult Comorbidity Evaluation–27.

**Supplemental Table 4**

| **Supplemental Table 4.** Association between patients’ characteristics and comorbidity. | | | | | | | | | |
| --- | --- | --- | --- | --- | --- | --- | --- | --- | --- |
|  | **Female** | **Male** | **p** | **Age (>median)** | **Age (<median)** | **p** | **Stage I/II** | **Stage II/IV** | **p** |
| CCI |  |  | **0.019** |  |  | **<0.0001** |  |  | 0.450 |
| CCI 0 | 141 | 452 |  | 233 | 360 |  | 192 | 373 |  |
| CCI 1 | 38 | 200 |  | 122 | 116 |  | 68 | 160 |  |
| CCI 2 | 16 | 92 |  | 81 | 27 |  | 37 | 64 |  |
| CCI 3+ | 27 | 128 |  | 111 | 44 |  | 54 | 92 |  |
| ACCI |  |  |  |  |  |  |  |  |  |
| ACCI 0-1 | 93 | 316 | 0.191 | NA |  |  | 117 | 250 | 0.289 |
| ACCI 2-3 | 62 | 241 |  |  |  |  | 129 | 264 |  |
| ACCI 4+ | 93 | 316 |  |  |  |  | 105 | 175 |  |
| HNCCI |  |  | **0.020** |  |  | **<0.0001** |  |  | 0.965 |
| HNCCI 0 | 151 | 504 |  | 274 | 381 |  | 211 | 414 |  |
| HNCCI 1 | 51 | 257 |  | 174 | 134 |  | 97 | 194 |  |
| HNCCI 2+ | 20 | 111 |  | 99 | 32 |  | 43 | 81 |  |
| SCS |  |  |  |  |  |  |  |  | 0.145 |
| SCS 0-1 | 140 | 358 | **<0.0001** | 249 | 249 | 1.000 | 169 | 299 |  |
| SCS 2+ | 82 | 514 |  | 298 | 298 |  | 182 | 390 |  |
| ACE-27 |  |  | **<0.0001** |  |  | **<0.0001** |  |  | 0.202 |
| ACE-27 grade 0 | 122 | 294 |  | 185 | 231 |  | 144 | 250 |  |
| ACE-27 grade 1 | 74 | 459 |  | 259 | 274 |  | 158 | 352 |  |
| ACE-27 grade 2 | 3 | 33 |  | 22 | 14 |  | 10 | 25 |  |
| ACE-27 grade 3 | 23 | 86 |  | 81 | 28 |  | 39 | 62 |  |
|  | **Smoking/No** | **Smoking/Yes** | **p** | **Alcohol/No** | **Alcohol/Yes** | **p** | **Anemia/Yes** | **Anemia/No** | **p** |
| CCI |  |  | 0.235 |  |  | **0.040** |  |  | **<0.0001** |
| CCI 0 | 381 | 212 |  | 420 | 173 |  | 98 | 485 |  |
| CCI 1 | 135 | 103 |  | 145 | 93 |  | 69 | 165 |  |
| CCI 2 | 65 | 43 |  | 69 | 39 |  | 22 | 83 |  |
| CCI 3+ | 97 | 58 |  | 104 | 51 |  | 60 | 94 |  |
| ACCI |  |  | **0.001** |  |  | **0.007** |  |  | **<0.0001** |
| ACCI 0-1 | 209 | 173 |  | 237 | 145 |  | 65 | 310 |  |
| ACCI 2-3 | 264 | 145 |  | 279 | 130 |  | 94 | 308 |  |
| ACCI 4+ | 205 | 98 |  | 222 | 81 |  | 90 | 209 |  |
| HNCCI |  |  | 0.060 |  |  | **0.003** |  |  | **<0.0001** |
| HNCCI 0 | 424 | 231 |  | 467 | 188 |  | 109 | 536 |  |
| HNCCI 1 | 181 | 127 |  | 194 | 114 |  | 93 | 208 |  |
| HNCCI 2+ | 73 | 58 |  | 77 | 54 |  | 47 | 83 |  |
| SCS |  |  |  |  |  | **<0.0001** |  |  | **0.001** |
| SCS 0-1 | NA |  |  | 452 | 46 |  | 89 | 399 |  |
| SCS 2+ |  |  |  | 286 | 310 |  | 160 | 428 |  |
| ACE-27 |  |  | **<0.0001** |  |  |  |  |  | **<0.0001** |
| ACE-27 grade 0 | 347 | 69 |  | NA |  |  | 61 | 349 |  |
| ACE-27 grade 1 | 236 | 297 |  |  |  |  | 137 | 387 |  |
| ACE-27 grade 2 | 20 | 16 |  |  |  |  | 10 | 23 |  |
| ACE-27 grade 3 | 75 | 34 |  |  |  |  | 41 | 68 |  |

NA= not applicable; CCI = Charlson Comorbidity Index; ACCI = age-adjusted CCI; HNCCI = Head neck CCI; SCS = simplified comorbidity scale; ACE-27 = Adult Comorbidity Evaluation–27

**Supplemental Table 5**

| **Supplemental Table 5.** Association between patients’ therapy and comorbidity. | | | | | | | | | |
| --- | --- | --- | --- | --- | --- | --- | --- | --- | --- |
|  | **Surgery/No** | **Surgery/Yes** | **p** | **Radiotherapy/No** | **Radiotherapy/Yes** | **p** | **Chemotherapy/No** | **Chemotherapy/Yes** | **p** |
| CCI |  |  | **0.053** |  |  | **<0.0001** |  |  | **<0.0001** |
| CCI 0 | 89 | 504 |  | 166 | 427 |  | 313 | 280 |  |
| CCI 1 | 44 | 194 |  | 67 | 171 |  | 132 | 106 |  |
| CCI 2 | 22 | 86 |  | 47 | 61 |  | 76 | 32 |  |
| CCI 3+ | 37 | 118 |  | 69 | 86 |  | 113 | 42 |  |
| ACCI |  |  | **0.002** |  |  | **<0.0001** |  |  | **<0.0001** |
| ACCI 0-1 | 48 | 334 |  | 84 | 298 |  | 181 | 201 |  |
| ACCI 2-3 | 75 | 334 |  | 127 | 282 |  | 224 | 185 |  |
| ACCI 4+ | 69 | 234 |  | 138 | 165 |  | 229 | 74 |  |
| HNCCI |  |  | **0.001** |  |  | **<0.0001** |  |  | **<0.0001** |
| HNCCI 0 | 92 | 563 |  | 186 | 469 |  | 352 | 303 |  |
| HNCCI 1 | 67 | 241 |  | 102 | 206 |  | 181 | 127 |  |
| HNCCI 2+ | 33 | 98 |  | 61 | 70 |  | 101 | 30 |  |
| SCS |  |  | 0.238 |  |  | 0.526 |  |  | 0.844 |
| SCS 0-1 | 80 | 418 |  | 154 | 344 |  | 287 | 211 |  |
| SCS 2+ | 112 | 484 |  | 195 | 401 |  | 347 | 249 |  |
| ACE-27 |  |  | 0.064 |  |  | 0.147 |  |  | **<0.0001** |
| ACE-27 grade 0 | 57 | 359 |  | 124 | 292 |  | 226 | 190 |  |
| ACE-27 grade 1 | 104 | 429 |  | 167 | 366 |  | 302 | 231 |  |
| ACE-27 grade 2 | 7 | 29 |  | 15 | 21 |  | 22 | 14 |  |
| ACE-27 grade 3 | 24 | 85 |  | 43 | 66 |  | 84 | 25 |  |
|  | **Cetuximab/No** | **Cetuximab/Yes** | **p** | **BSC/No** | **BSC/Yes** | **p** | **Single modality*** | **Multiple modalities*** | **p** |
| CCI |  |  | 0.191 |  |  | **0.001** |  |  | **<0.0001** |
| CCI 0 | 504 | 89 |  | 583 | 10 |  | 170 | 413 |  |
| CCI 1 | 197 | 41 |  | 238 | 0 |  | 77 | 161 |  |
| CCI 2 | 96 | 12 |  | 102 | 6 |  | 46 | 56 |  |
| CCI 3+ | 139 | 16 |  | 146 | 9 |  | 72 | 74 |  |
| ACCI |  |  | 0.101 |  |  | **0.001** |  |  | **<0.0001** |
| ACCI 0-1 | 315 | 67 |  | 378 | 4 |  | 84 | 294 |  |
| ACCI 2-3 | 356 | 53 |  | 403 | 6 |  | 137 | 266 |  |
| ACCI 4+ | 265 | 38 |  | 288 | 15 |  | 144 | 144 |  |
| HNCCI |  |  | 0.420 |  |  | **<0.0001** |  |  | **<0.0001** |
| HNCCI 0 | 558 | 97 |  | 645 | 10 |  | 190 | 455 |  |
| HNCCI 1 | 261 | 47 |  | 303 | 5 |  | 114 | 189 |  |
| HNCCI 2+ | 117 | 14 |  | 121 | 10 |  | 61 | 60 |  |
| SCS |  |  | 0.232 |  |  | 0.170 |  |  | 0.699 |
| SCS 0-1 | 433 | 65 |  | 490 | 8 |  | 164 | 326 |  |
| SCS 2+ | 503 | 93 |  | 579 | 17 |  | 201 | 378 |  |
| ACE-27 |  |  | 0.257 |  |  | 0.544 |  |  | **0.003** |
| ACE-27 grade 0 | 363 | 53 |  | 410 | 6 |  | 128 | 282 |  |
| ACE-27 grade 1 | 449 | 84 |  | 518 | 15 |  | 170 | 348 |  |
| ACE-27 grade 2 | 28 | 8 |  | 35 | 1 |  | 15 | 20 |  |
| ACE-27 grade 3 | 96 | 13 |  | 106 | 3 |  | 52 | 54 |  |

BSC= best supportive care; *BSC patients excluded; CCI = Charlson Comorbidity Index; ACCI = age-adjusted CCI; HNCCI = Head neck CCI; SCS = simplified comorbidity scale; ACE-27 = Adult Comorbidity Evaluation–27

**Supplemental Table 6**

| **Supplemental Table 6.** Association of baseline parameters and of comorbidity on overall survival (N = 1,094 patients) | | | | |
| --- | --- | --- | --- | --- |
| **Parameter** | **Dichotomized** | **2-year**  **OS** | **5-year**  **OS** | **log Rank p** |
| All |  | 81.1 | 71.0 |  |
| Gender | Male | 80.0 | 69.6 | 0.052 |
|  | Female | 85.7 | 77.0 |  |
| Age (years; median: 60.3) | <Median | 83.7 | 74.3 | **0.019** |
|  | >Median | 78.4 | 67.2 |  |
| Site | Larynx | 88.6 | 74.5 | **0.016** |
|  | Other | 79.3 | 70.5 |  |
| UICC stage | I-II | 91.3 | 83.6 | **<0.0001** |
|  | III-IV | 75.1 | 63.3 |  |
| Histology | SCC | 80.2 | 69.7 | **0.042** |
|  | Other | 88.1 | 81.6 |  |
| Grading | G1/2 | 83.0 | 71.3 | 0.074 |
|  | G3/4 | 76.4 | 69.7 |  |
| Surgery | No | 61.6 | 46.0 | **<0.0001** |
|  | Yes | 84.7 | 75.0 |  |
| Radiotherapy | No | 84.7 | 77.7 | **0.061** |
|  | Yes | 79.6 | 67.9 |  |
| Chemotherapy | No | 84.9 | 76.8 | **0.002** |
|  | Yes | 76.5 | 63.5 |  |
| Cetuximab | No | 68.0 | 43.1 | **NA** |
|  | Yes | 83.1 | 74.6 |  |
| Only best supportive care | No | 81.9 | 71.7 | **<0.0001** |
|  | Yes | 26.9 | NA |  |
| Smoker | No | 84.6 | 76.6 | **<0.0001** |
|  | Yes | 75.4 | 61.2 |  |
| Alcohol | No | 85.4 | 76.0 | **<0.0001** |
|  | Yes | 72.2 | 60.0 |  |
| Anemia | No | 84.5 | 73.8 | **<0.0001** |
|  | Yes | 69.8 | 61.3 |  |
| CCI | CCI 0-1 | 83.5 | 76.2 | **<0.0001** |
|  | CCI 2+ | 73.1 | 52.9 |  |
| ACCI | ACCI 0-3 | 83.8 | 75.6 | **<0.0001** |
|  | ACCI 4+ | 73.4 | 56.5 |  |
| HNCCI | HNCCI 0-1 | 82.7 | 73.8 | **<0.0001** |
|  | HNCCI 2+ | 67.9 | 45.3 |  |
| SCS | SCS 0-1 | 87.2 | 79.7 | **<0.0001** |
|  | SCS 2+ | 75.9 | 63.4 |  |
| ACE-27 | ACE-27 grade 0-1 | 88.4 | 82.7 | **<0.0001** |
|  | ACE-27 grade 2-3 | 76.6 | 63.2 |  |

OS = overall survival; SCC = squamous cell carcinoma; NA = not applicable; CCI = Charlson Comorbidity Index; ACCI = age-adjusted CCI; HNCCI = Head neck CCI, SCS = simplified comorbidity scale; ACE-27 = Adult Comorbidity Evaluation–27.

**Supplemental Table 7**

| **Supplemental Table 7.** Comparison of the present study to other **population-based studies** on the impact of comorbidity on overall survival in head and neck cancer | | | | | | | | |
| --- | --- | --- | --- | --- | --- | --- | --- | --- |
| **Study** | **Country, source** | **Year of diagnosis** | **Number of patients, therapy** | **Tumor site** | **Prevalence of comorbidity** | **5-year OS related to CCI/ACE-27** | **Smoking, alcohol, HPV** | **Comment** |
| Present study | Germany, cancer registry | 2009 to 2011 | 1,094 patients with different types of therapy | Head and neck carcinoma, all M0 | CCI >0: 46%  ACCI>0: 89%  HNCCI>0: 40%  SCS>0: 65%  ACE-27>0 62% | CCI=0: 78%  CCI=1: 71%  CCI=2: 49%  CCI=3+: 41%  ACE-27 0: 83%  ACE-27 1: 63%  ACE-27 2: 56%  ACE-27 3: 64% | Smoking: 38%  Drinking: 33%  HPV: unknown |  |
| Yang et al. [[11](#_ENREF_11)] | Taiwan, national database | 2007 to 2011 | 4,095 patients with curative radiotherapy | Nasopharyngeal carcinoma, all M0 | CCI >0: 60%  ACCI>0: 60%  HNCCI>0: 25% | CCI=0: 77%  CCI=1-5: 63%  CCI6+ 40% | Smoking: unknown  Drinking: unknown  HPV: unknown | no TNM staging information; ACCI most appropriate index |
| Boje et al. ,[[10](#_ENREF_10)] | Denmark, DAHANCA database | 1992 to 2008 | 12,623 patients with all types of therapy, but mainly radiotherapy | Head and neck carcinoma, all TNM stages | CCI>0: 36% | CCI=0: 50%  CCI=1: 45%  CCI=2: 39%  CCI=3+: 33% | Smoking: unknown  Drinking: unknown  HPV: unknown | Cancer specific death was not affected by comorbidity |
| Boje et al. [[18](#_ENREF_18)] | Denmark | 1992 to 2008 | 9,388 patients with curative radiotherapy | Larynx, oral cavity, pharynx, all M0 | CCI>0: 36%  HNCCI>0: 27% | CCI=0: 50%  CCI=1: 43%  CCI=2: 40%  CCI=3+: 34% | Smoking: unknown  Drinking: unknown  HPV: unknown | Study performed to establish the HNCCI |
| Piccirillo et al. [[20](#_ENREF_20)] | United, States, SEER | 1995 to 1996 | 341 patients, therapy data not given | Head and neck carcinoma, all TNM | ACE-27 >0: 45% | Only 2y-OS given!  ACE-27 0: 82%  ACE-27 1: 82%  ACE-27 2: 55%  ACE-27 3: 0% | Smoking: unknown  Drinking: unknown  HPV: unknown | With comparison to , colorectum, lung, breast  other gynecological sites, or prostate cancer |
| Landis et al. [[9](#_ENREF_9)] | Netherlands | 2000 to 2006 | 1,499 patients with different types of therapy | Larynx, oral cavity, oropharynx, hypopharynx, all TNM | no comorbidity index calculated  most frequent comorbidity:  cardiovascular: 41% | NA | Smoking: unknown  Drinking: unknown  HPV: unknown | Comparison to cancer-free controls |
| Rose et al. [[8](#_ENREF_8)] | United States, SEER | 1994 to 2003 | 34,568 patients with different types of therapy | oral cavity, nasopharynx, oropharynx, hypopharynx, larynx, all M0 | no comorbidity index calculated  most frequent comorbidity:  cardiovascular: 28% | NA | Smoking: unknown  Drinking: unknown  HPV: unknown | Multiple factors affect risk of competing mortality |
| Genther et al. [[5](#_ENREF_5)] | United States, NIS | 2001 to 2010 | 61,741 patients with different types of surgical therapy | >65 years of age; oral cavity, oropharynx, hypopharynx, larynx, all M0 | CCI>0: 50% | NA | Smoking: 12%  Drinking: 5%  HPV: unknown | Advanced comorbidity in surgical patients  is associated with increased mortality, morbidity, length  of hospitalization. |
| Piccirillo et al. [[38](#_ENREF_36)] | United States, SEER | 1983 to 1994 | 15,493 patients with different types of therapy | oral cavity, pharynx, larynx, all TNM | CCI>0: 65% | CCI=0: 45%  CCI=1-2 39%  CCI=3+: 34% | Smoking: unknown  Drinking: unknown  HPV: unknown | No therapy information; WUHNCI and HNCA also applied. Comorbidity weak predictor |
| Reid et al. [[23](#_ENREF_23)] | United States, SEER | 1985 to 1993 | 9,386 patients with all types of therapy | oral cavity, pharynx, larynx, all TNM | CCI>0: 12% | NA | Smoking: unknown  Drinking: unknown  HPV: unknown | Also HNCA and ATC index used. CCI and HNCA better predictive value |
| Reid et al. [[39](#_ENREF_37)] | United States, HCFA SEER | 1985 to 1993 | 9,386 patients with all types of therapy | >65 years of age; oral cavity, pharynx, larynx, all TNM | CCI>0: 12% | NA | Smoking: unknown  Drinking: unknown  HPV: unknown | comorbidity predictor of survival also in elderly head neck cancer population |
| Schwam et al. [[24](#_ENREF_24)] | Unites States, NCDB | 2003 to 2006 | 2,525 patients with all types of therapy | oral cavity, pharynx, larynx, all TNM | CCI>0: 23% | NA | Smoking: unknown  Drinking: unknown  HPV: unknown | comorbidity no predictor for therapy decision |

ACE-27 = Adult Comorbidity Evaluation–27; ATC = alcohol- and tobacco-related comorbidity index; DAHANCA = Danish Head and Neck Cancer group; CCI = Charlson Comorbidity Index; ACCI = age-adjusted CCI; HNCCI = Head neck CCI, SCS = simplified comorbidity scale; OS =overall survival; HPV = human papillomavirus; SEER = Surveillance, Epidemiology, and End Results database; NCDB = National Cancer Database; WUHNCI=Washington University Head and Neck Index; HNCA = Head and Neck Cancer Index; NIS = Nationwide Inpatient Sample (NIS), Healthcare Cost and Utilization Project, Agency for Healthcare Research and Quality (AHRQ); HCFA = Health Care Finance Administration Medicare.

**Supplemental Table 8**

| **Supplemental Table 8.** Comparison of the present study to other **larger hospital-based studies (>100 patients)** on the impact of comorbidity on overall survival in head and neck cancer | | | | | | | | |
| --- | --- | --- | --- | --- | --- | --- | --- | --- |
| Lee et al [[40](#_ENREF_38)] | Taiwan | 2006 to 2011 | 232 patients with surgical therapy | Oral cancer, all M0 | CCI>0: 27% | Only 2y-OS given!  CCI=0-1: 80%  CCI=2+ 70% | Smoking: unknown  Drinking: unknown  HPV: unknown | Also Elixhauser score used. CCI was not an independent prognosticator |
| Yung and Piccirillo [[41](#_ENREF_39)] | United States | 1997 to 1998 | 183 patients with all types of therapy | oral cavity, oropharynx, larynx, all TNM | ACE-27>0: 68% | NA | Smoking: unknown  Drinking: 4%  HPV: unknown | 32% change of comorbidity after treatment |
| Datema et al. [[7](#_ENREF_7)] | Netherlands | 1981 to 1998 | 1,371 patients with all types of therapy | Head and neck carcinoma, all TNM | ACE-27>0 36% | ACE-27 0: 58%  ACE-27 1: 53%  ACE-27 2: 44%  ACE-27 3: 25% | Smoking: unknown  Drinking: unknown  HPV: unknown | Impact of ACE27 grade 3 is comparable to the impact of a T4 tumor or an N2 neck |
| Sanabria et al. [[42](#_ENREF_40)] | Brazil | 1990 to 2003 | 310 patients with all types of therapy | > 70 years of age; Head and neck carcinoma, all TNM | ACE-27>0 75% | ACE-27 0: 68%  ACE-27 1: 57%  ACE-27 2: 75%  ACE-27 3: 61% | Smoking: 41%  Drinking: 39%  HPV: unknown | Comorbidity also relevant in older patients |
| Ankola et al. [[43](#_ENREF_41)] | United States | 2002 to 2011 | 288 patients with all types of therapy | Head and neck carcinoma, all TNM | ACE-27>0 81% | ACE-27 0-1: 65%  ACE-27 2-3: 55% | Smoking: 40%  Drinking: 27%  HPV: 10% | HPV+ patients had lower comorbidity |
| Mell et al. [[12](#_ENREF_12)] | United States | 1993 to 2005 | 479 patients with radio- chemotherapy | only stage III/IV form organ-preservation radiochemotherapy protocols | CCI>0: 49% | NA | Smoking: 67%  Drinking: 61%  HPV: unknown | Comorbidity leads to competing mortality |
| Kwon et al. [[44](#_ENREF_42)] | Korea | 2001 to 2010 | 600 patients with all types of therapy | only stage III/IV oral cavity, oropharynx,  larynx, hypopharynx, M0 | CCI>0: 44% | NA | Smoking: 43%  Drinking: 12%  HPV: unknown | Comorbidity leads to competing mortality and non-cancer health events |
| Peters et al. [[45](#_ENREF_43)] | Netherlands | 1995 to 2010 | 1,201 patients with surgical treatment | Head and neck carcinoma, all M0 | NA | NA | Smoking: unknown  Drinking: unknown  HPV: unknown | Specific comorbidities are associated with  specific postoperative complications |
| Connor et al. [[46](#_ENREF_44)] | Scotland | 1999 to 2010 | 137 patients with all types of therapy | stage III laryngeal cancer | ACE-27>0: 81% | NA | Smoking: 51%  Drinking: 16%  HPV: unknown | Severe comorbidity was associated with selection bias to radiotherapy |
| Omura et al. [[47](#_ENREF_45)] | Japan | 2004 to 2011 | 185 patients with surgical treatment | stage III/IV, head and neck carcinoma, all M0 | ACE>27>0: 61% | ACE-27 0-1: 60%  ACE-27 2-3: 24% | Smoking: 51%  Drinking: 2%  HPV: unknown | Comorbidity also predicted distant failure |
| Baijal et al. [[48](#_ENREF_46)] | India | 2 months in 2010 | 200 patients with radiotherapy | Head and neck carcinoma, all TNM | CCI>0: 18%  ACE-27>0: 32% | NA | Smoking/Drinking:  73%  HPV: unknown | Comorbidity can influence decision making |
| Deneuve et al. [[49](#_ENREF_47)] | France | 1999 to 2007 | 111 patients with all types of therapy | only obese patients, head and neck carcinoma, all TNM | ACE-27>0: 77% | NA | Smoking: 83%  Drinking: 44%  HPV: unknown | Obesity not risk factor for OS |

ACE-27 = Adult Comorbidity Evaluation–27; ATC = alcohol- and tobacco-related comorbidity index; DAHANCA = Danish Head and Neck Cancer group; CCI = Charlson Comorbidity Index; ACCI = age-adjusted CCI; HNCCI = Head neck CCI, SCS = simplified comorbidity scale; OS =overall survival; HPV = human papillomavirus; SEER = Surveillance, Epidemiology, and End Results database; WUHNCI=Washington University Head and Neck Index; HNCA = Head and Neck Cancer Index; NIS = Nationwide Inpatient Sample (NIS), Healthcare Cost and Utilization Project, Agency for Healthcare Research and Quality (AHRQ); HCFA = Health Care Finance Administration Medicare.

References

38. Piccirillo JF, Spitznagel EL, Jr., Vermani N, Costas I, Schnitzler M. Comparison of comorbidity indices for patients with head and neck cancer. *Med Care* 2004;42:482-6.

39. Reid BC, Alberg AJ, Klassen AC, Samet JM, Rozier RG, Garcia I, et al. Comorbidity and survival of elderly head and neck carcinoma patients. *Cancer*. 2001;92:2109-16.

40. Lee CC, Ho HC, Su YC, Chen PC, Yu CH, Yang CC. Comparison of different comorbidity measures for oral cancer patients with surgical intervention: A longitudinal study from a single cancer center. *Auris Nasus larynx.* 2016;43:322-9.

41. Yung KC, Piccirillo JF. The incidence and impact of comorbidity diagnosed after the onset of head and neck cancer. Arch Otolaryngol Head Neck Surg 2008;134:1045-9.

42. Sanabria A, Carvalho AL, Vartanian JG, Magrin J, Ikeda MK, Kowalski LP. Comorbidity is a prognostic factor in elderly patients with head and neck cancer. *Ann Surg Oncol* 2007;14:1449-57.

43. Ankola AA, Smith RV, Burk RD, Prystowsky MB, Sarta C, Schlecht NF. Comorbidity, human papillomavirus infection and head and neck cancer survival in an ethnically diverse population. *Oral Oncol* 2013;49:911-7.

44. Kwon M, Roh JL, Song J, Lee SW, Kim SB, Choi SH, et al. Noncancer health events as a leading cause of competing mortality in advanced head and neck cancer. *Ann Oncol* 2014;25:1208-14.

45. Peters TT, van Dijk BA, Roodenburg JL, van der Laan BF, Halmos GB. Relation between age, comorbidity, and complications in patients undergoing major surgery for head and neck cancer. *Ann Surg Oncol* 2014;21:963-70.

46. Connor KL, Pattle S, Kerr GR, Junor E. Treatment, comorbidity and survival in stage III laryngeal cancer. *Head Neck*. 2015;37:698-706.

47. Omura G, Ando M, Saito Y, Kobayashi K, Yamasoba T, Asakage T. Comorbidity as predictor poor prognosis for patients with advanced head and neck cancer treated with major surgery. *Head Neck* 2016;38:364-9.

48. Baijal G, Gupta T, Hotwani C, Laskar SG, Budrukkar A, Murthy V, et al. Impact of comorbidity on therapeutic decision-making in head and neck cancer: audit from a comprehensive cancer center in India. *Head Neck* 2012;34:1251-4.

49. Deneuve S, Tan HK, Eghiaian A, Temam S. Management and outcome of head and neck squamous cell carcinomas in obese patients. *Oral Oncol* 2011;47:631-5.
